# Students with hearing impairment at a South African university: Self-identity and disclosure

**DOI:** 10.4102/ajod.v5i1.229

**Published:** 2016-09-22

**Authors:** Diane Bell, Arend Carl, Estelle Swart

**Affiliations:** 1 School of Business, University of Stellenbosch, South Africa; 2 Faculty of Education, University of Stellenbosch, South Africa

## Abstract

**Background:**

A growing number of students with hearing loss are being granted access to higher education in South Africa due to the adoption of inclusive educational policies. However, available statistics indicate that participation by students with hearing impairments in higher education remains low and research suggests that support provisioning for those who do gain access is inadequate.

**Objectives:**

This article aims to illustrate that the assumed self-identity of students with hearing impairment influences their choice to disclose their disability. The choice not to disclose their hearing loss prevents them from accessing the necessary reasonable accommodations and this in turn may affect their eventual educational success.

**Method:**

Reported here is a qualitative descriptive case study at a South African university. Purposive sampling methods were employed. Data were gathered from in-depth interviews with seven students with hearing impairment ranging from moderate to profound, using spoken language. Constructivist grounded theory was used as an approach to the process of generating and transforming the data, as well as the construction of theory.

**Findings:**

All the student participants identified as having a hearing rather than a D/deaf identity cultural paradigm and viewed themselves as ‘normal’. Linked to this was their unwillingness to disclose their hearing impairment and thus access support.

**Conclusion:**

It is crucially important for academic, support and administrative staff to be aware of both the assumed ‘hearing’ identity and therefore subsequent non-disclosure practices of students with a hearing impairment using the oral method of communication. Universities need to put measures in place to encourage students to voluntarily disclose their hearing impairment in order to provide more targeted teaching and learning support. This could lead to improved educational outcomes for students.

## Introduction

Hearing impairment is recognised as a global pandemic (Tucci, Merson & Wilson 2009) and it is also the most common congenital abnormality found in newborns (Shemesh 2010). In South Africa, Stats SA, using the Washington Group Model, estimates an impairment prevalence of 7.5% derived from the 2011 National Census. The results from the same census (Statistics South Africa 2011) related to hearing impairment revealed that 0.1% of the population ‘cannot hear at all’, 0.5% experience ‘a lot of difficulty’, 2.9% experience ‘some difficulty’ and the balance 96.4% have ‘no difficulty’ hearing.

Since 1994, the South African government has been committed to the transformation of the entire education system with changes in global initiatives regarding inclusive education, also influencing the drive towards inclusion locally (Naicker 2000; United Nations Convention on the Rights of Persons with Disabilities 2006). Therefore, higher education institutions (HEIs) have been encouraged to promote both equal access and participation to all students, irrespective of race, gender, language, age or disability (Department of Education 2001). More recently, the South African government has released the Draft National Disability Rights Policy (Department of Social Development [DSD] 2015a), with the express purpose of establishing a policy framework that provides coherence to and guides government activity across disability-strategic areas of public policy and programmes as well as the White Paper on the Rights of Persons with Disabilities (WPRPD) (DSD 2015b), which aims to accelerate transformation and redress by promoting full inclusion, integration and equality for persons with disabilities. The vision of the WPRPD is the domestication of the United Nations Convention on the Rights of Persons with Disabilities (UNCRPD) and the creation of a free and just society, inclusive of all persons with disabilities (DSD 2015b). Both these documents could serve to promote the rights of all students in higher education.

Despite students with disabilities being increasingly granted access to higher education, it is disturbing to note that, according to a survey conducted by Crous (2004:228) at three universities in South Africa, it was found that less than 0.5% of the student population was represented by students with disabilities. More recent data from 22 of the 23 public universities showed that 5 807 students with disabilities were enrolled in HEIs in 2011, accounting for only 1% of the total enrolment (Foundation of Tertiary Institutes of the Northern Metropolis [FOTIM] 2011). It is also interesting to note that, although levels of support vary from university to university, the support is mainly provided to those students with mobility and visual impairments (FOTIM 2011). As per the WPRPD (DSD 2015b), most young adults aged 20–24 years with severe difficulties across all functional domains were not attending any tertiary institution. The white paper further reports that only one-fifth of persons with severe difficulties were attending any tertiary institution.

Statistics in South Africa regarding the numbers of students who have disclosed disabilities, and more specifically hearing impairment, are not readily available due to factors such as differing definitions of disability, misinterpretation of disability codes on university application forms and stigma associated with disclosure of a disability (De Cesarei 2014). Higher Education Management Information System (HEMIS) data obtained from the Department of Higher Education and Training (DHET) for the period 2003–2010 indicated a growth in the prevalence of students with hearing impairment registered at HEIs in South Africa from only 155 in 2003 to 326 in 2010.

Despite this growth, it is clear that students with hearing impairment remain completely under-represented and under-supported in higher education, in both developed and developing countries (Brett 2010:5; Higher Educational Statistical Agency 2011). Many reasons may be offered for this under-representation, such as the needs of students with hearing impairment being unique to each individual student, their support needs being complex due to communication barriers and the cost of support provisioning, such as for (human) note-takers and interpreters.

Little is known about how students with hearing impairment experience higher education (Lang 2002; Luckner, Slike & Johnson 2012). However, what is known is that of those students who do enter higher education, many do not graduate successfully due to a variety of factors, such as lack of support, specifically for students with hearing impairments. The academic achievement gap between students who hear and those with a hearing impairment is a frequently reported fact (Marschark 2006; Meadow-Orleans 2001; Moores 2003). In South Africa, almost no research has been conducted concerning students with hearing impairment (using the oral method of communication) in higher education, especially regarding their teaching and learning needs (FOTIM 2011). Previous South African studies have mostly focused on teacher training (of the Deaf), early hearing detection and intervention, development of a Deaf identity and Sign Language and Deaf adults’ views on Deaf Education in South Africa (McIlroy & Storbeck 2011).

Note the use of terminology: Hearing Impairment refers to a condition in persons with varying degrees of hearing loss not using South African Sign Language (SASL) as a primary medium of communication, who use various means of communication and assistive hearing technologies. These include speech, speech/lip reading, hearing aid systems, cochlear implants, bone anchored hearing aid and applicable assistive listening devices, etc., or a combination thereof (Nair 2015).

The issue of identity is crucial for academic success as students’ self-perception and their perception of how others view them play a pivotal role in students’ interactions with both institutional processes and structures, which has important implications for accessing support through personal disclosure as well as teaching and learning experiences, and possibly educational outcomes. Due to the invisibility of hearing impairment, the assumed identity of the student with a hearing impairment is open to perpetual negotiation (‘normal hearing’ versus having a disability). By avoiding confronting their hearing impairment through self-disclosure and seeking reasonable accommodations, these students may not be able to enjoy full and equal participation in academic life (Hindhede 2011).

This article aims to illustrate that the assumed self-identity of students with hearing impairment influences their choice to disclose their disability. This choice may in turn negatively affect their eventual educational success. It is important for academic, support and administrative staff to be aware of both the assumed identity and non-disclosure practices of students with a hearing impairment using the oral method of communication in order to put measures in place to encourage disclosure and to provide more targeted teaching and learning support.

The next few sections of this article will focus firstly on the theoretical perspectives and then the empirical study. The theoretical aspects are:

- an exploration of the constructs of disability as these constructs tend to frame our thinking and thus our responses to persons with disabilities;
- the two main ways in which deafness is conceptualised;
- how the assumed identity of a student with a hearing impairment as either Culturally Deaf or Hearing contributes to positive aspects of his or her social identity and finally;
- how this can be measured via the Deaf Identity Development Scale (DIDS) (Glickman & Carey 1993).

## Disability – changing paradigms

Throughout history, changes in society have frequently been paralleled by new ways of thinking or new paradigms. Over the last 20 years, there have been challenges to dominant perceptions of and attitudes to people with disabilities (Council on Higher Education 2005) as well as a great deal of discussion about different theoretical approaches to disability. In the Western world, the history of disability has been characterised by the progressive development of various models of disability, with the medical/genetic model (Oliver 1996) and the social model (Shakespeare 2006) (currently viewed as the dominant model by disability activists and academics within higher education globally) being most prominent. These models or constructs of disability have set the parameters for society’s response to people with disabilities, framing our thinking and way of living. In a recent article by Luckner *et al*. (2012:59), five specific challenges that often occur as by-products of a hearing impairment and that interfere with typical ways of learning are presented, namely: language, vocabulary and literacy delays; gaps in background and domain knowledge; inadequate knowledge and use of learning strategies; social skills deficits; and reliance on assistive technologies.

In South Africa, students with hearing impairment face a multitude of barriers in higher education. There could be many reasons to explain why these barriers exist, such as lack of support, lack of awareness of the accommodation needs of these students, the ‘invisibility’ of their hearing impairment, the uniqueness of hearing impairment and therefore complex support needs, teaching faculty ignoring calls for attendance at disability-related professional development courses and lack of financial and human resources, to mention but a few. These factors make it ‘unattractive’ to universities to admit students with hearing impairments, resulting in under-representation in higher education. Furthermore, these barriers, as reported by Howell (2006:170), have a profound and sustained effect on the psychosocial well-being and functioning of the student. Disabled people who have managed to attend HEIs argue that the energy, emotional resources and levels of stress involved in dealing with the overwhelming range of barriers that confront them are extremely undermining and place them at an ongoing disadvantage in terms of other students, and if they are unable to ‘deal with’ these issues, the prevailing attitudes and prejudices towards their abilities are reinforced.

It is important to address these barriers to ensure educational success for students with hearing impairments. If not, these students are more likely to be excluded from participation, more likely to require services to enable their participation and more likely to self-identify in questions relating to disability status.

In South Africa, the very limited response by universities to students with hearing impairment occurs in the form of various types of teaching and learning support, such as preferential seating, extra writing time, hearing augmentation devices (e.g. hearing loops) and note-takers. As a move away from the previously dominant medical model (where disability could be medically ‘fixed’), the social model of disability (where persons with disabilities are ‘disabled’ by society) is firmly entrenched (more in words than in practice) in higher education in South Africa (CHE 2005). Although support services for students with disabilities are currently based on this model, it seems that Reindal’s social-relational model (2008) could be more suited to inclusion as it retains the main tenets of the social model of disability, such as the effects of impairment and the phenomenon of disability, elaborated as a ‘social relational phenomenon’ but it also maintains the perspective of ‘oppression and discrimination’ in contrast to ‘disadvantage due to restriction of activity’ (Reindal 2008:143). Using this model, one can equally incorporate the personal experiences of persons living with reduced functions, both socially and without adopting the individualistic or medical model.

In terms of this social-relational model, a person with reduced hearing function would be referred to as being ‘hearing impaired’ (in line with the social model), but additionally, his or her personal experiences of being a person living with reduced function plus discrimination and oppression would be encapsulated (Bell 2013). The findings in the present study, with respect to identity, stigma and disclosure, were thus viewed through this model (as a lens) as the social-relational model works towards taking the ideals of inclusion forward, which could have a significant effect on educational attainment, especially for students with hearing impairment in South Africa. The following section explores the two main ways in which deafness is conceptualised.

## Disability versus linguistic minority

In society, there are two main constructions of deafness: one construes ‘deaf’ as a category of disability while the other construes ‘Deaf’ as being a member of a linguistic minority group with its associated culture (Lane 1994). Large disparities exist between these two groups, with the two constructions residing at opposite ends of a continuum. This, unfortunately, in the South African context, has been to the detriment of all persons who have a hearing impairment as there is no one organisation advocating for their rights and thus little progress has been made. Each of the D/deaf constructions (‘deaf’ as a category of disability versus ‘Deaf’ as linguistic minority group with their associated culture) has a core client group. The struggle between these two groups has endured for centuries (Lane 1994), in part because there is no simple criterion for identifying most childhood candidates as clients of one position or the other. It is generally accepted that if a hearing adult becomes deaf from illness or aging (develops a hearing impairment), then that person has a disability and he or she is not regarded as a member of Deaf culture (Lane 1994). The same is true of Deaf parents insisting that their Deaf child is part of the linguistic minority group. However, if a child with a profound hearing impairment is born to hearing parents and they choose cochlear implantation, their choice and membership are rejected by the Deaf community. In this study, the construction of ‘deafness’ was considered as a category of disability, which has an effect on the identity of the person with a hearing impairment who typically identifies as being ‘culturally hearing’. The next section explores social identity theory as it relates to the adoption of a specific type of identity by students with hearing impairment, which affects their choice to disclose their disability or not.

### Social identity theory

According to social identity theory (Tajfel 1981), an individual will remain a member of a group if that group contributes to positive aspects of his or her social identity, such as self-esteem. Bat-Chava (2000:420) argues that, through the route of individual mobility, ‘deaf’ people may assume a culturally hearing identity, assimilating as much as possible into the hearing world by using their residual hearing (either through amplification or cochlear implants) and speech-reading often resulting in a positive social identity through academic and professional accomplishment. The selection of a culturally hearing identity is often evident when students with hearing impairments attempt to assimilate fully and push themselves to overachieve in mainstream environments (Bat-Chava 2000). They tend to work much harder than their hearing peers to perform, with successful achievement, which in turn builds their self-esteem (Bat-Chava 2000). It is my contention that one’s personal construction of disability is a multifarious phenomenon, resulting in people with disabilities often having complex identities, seeing themselves as ‘normal’ and with limited identification with their hearing impairment.

The choice of cochlear implantation also affects identity. This is an emotive topic as some critics view the choice of surgical implantation as a cure – trying to become ‘normal’ and to being in denial concerning one’s disability (Sparrow 2005). A key issue in the debate about the appropriateness of implantation for children with hearing impairments has been around the notion of identity – where the individual ‘fits in’. Wald and Knutson (2000:89) questioned a group of 45 adolescents with and without cochlear implants regarding issues of Deaf identity and concluded that the groups were similar in many respects, but that the cochlear-implanted group rated hearing identity as a desirable goal more favourably than the non-implanted group. The authors attribute this to the audiological benefit that the implanted group received. In another study by Wheeler *et al*. (2007:314), some participants commented on the fact that, because of their good spoken language skills, and in particular, speech intelligibility, they were sometimes perceived as hearing by people who did not know them well. Wheeler *et al*. (2007) further found that the majority of the participants recognised themselves as intrinsically deaf (having a hearing impairment) in the sense that they could not hear without their implant but they did not demonstrate a culturally deaf identity. The following section explores the continuum of the DIDS.

### Deaf identity development scale

In 1993, Glickman and Carey developed an instrument, the DIDS to measure how deaf people identify with the Deaf community and Deaf culture. Along the continuum, four kinds of Deaf cultural identities, presumed to be developmentally related, are provided. The first kind of identity is called ‘culturally hearing’, which refers to the dominant ‘hearing’ understanding of deafness as a medical pathology or disability. The second kind of Deaf cultural identity is called ‘culturally marginal’. This orientation is typical of people who experience themselves as fitting between the Deaf and hearing worlds, comfortable in neither. The third kind of Deaf cultural identity is called ‘immersion’. This is relevant to the period when Deaf people immerse themselves in the Deaf world. The last kind of Deaf cultural identity is called ‘bicultural’, which means they are comfortable in both worlds.

According to this scale, students with a hearing impairment and who make use of oral communication could be considered, in terms of identity, as ‘culturally hearing’ – their hearing loss seen as a disability. Often, according to Hindhede (2011), in order to avoid embarrassment, the culturally hearing group pretend that they have heard what has been said and in order to avoid any awkward exposure and in an attempt to ‘be normal’, they refrain from requesting any accommodations that would help facilitate communication. They also develop what they perceive, from their point of view, to be perfectly adequate coping strategies in an attempt to be viewed as hearing.

The following section will describe the method used, provide a rational for the selection of the case and offer participant details.

## Research methodology

This research comprised a qualitative descriptive case study which sought to ensure that the topic of interest was well explored and that the essence of the phenomenon was revealed. The context was a South African university and the units of analysis were students with hearing impairment, their lecturers and the head of the disability unit. A constructivist paradigm was employed, which assumes that reality is socially constructed (Charmaz 2006). This implies that there is no single reality, but that each single event is interpreted through multiple realities (Merriam 2009:9). Through the subjective experiences shared by participating students, an understanding of how they, as a ‘bounded system’ (Merriam 2009), constructed their own meaning of their personal identity emerged. The present study was also descriptive in nature, describing the experiences of being students with a hearing impairment in a ‘hearing’ university. Their social worlds were explored, using both the participants’ and the researchers’ understandings (Ritchie & Lewis 2003). As a case study researcher, I was also able to use my experiences as a mother of a daughter with a profound hearing impairment, a lecturer, my involvement in the disability sector, but most importantly, the contextual accounts of the participants to assist me in the construction of knowledge.

One South African university was chosen as the context for the cases to be studied for the following reasons:

- HEMIS data (DHET 2010) indicated that the selected university had 43 students with disclosed hearing impairments in 2008 (from 15 in 2007) and was therefore selected on account of this high number of enrolments.
- This particular university has been supporting students with hearing impairment for the past few years and should therefore have gained some knowledge and experience in supporting them.
- Only one university was selected as a single case, rather than multiple universities as multiple cases, as each institution brings with it its own identity, culture, historical context and varying support for students with disabilities, particularly students with hearing impairments. Due to newborn screening and other active early identification programmes, children are fitted with hearing instruments (including cochlear implants) at a young age, which significantly impacts language development, but this is regionally dependent. Thus, student communities from the different universities in South Africa would differ significantly.

### Selecting and describing the participants

A purposeful sampling procedure (Patton 2002; Silverman 2010) was used to select the sample. The criteria for inclusion of students in the study were that participants:

- had to have hearing impairments, regardless of the degree of hearing impairment or the age of onset;
- needed to be registered students at the selected university (either undergraduate or postgraduate); and
- had to make use of spoken language (either English or Afrikaans as their home language) rather than Sign Language.

With the required permission obtained, students who had disclosed their hearing loss to the disability unit at the selected university were invited by email to participate in the research. Seven out of a possible 13 students volunteered to participate in the study (refer to Table 1).

**TABLE 1:** Biographical data for each student participant.

Participant (Pseudonyms have been used)	Age	Gender	Year of study	Onset	Degree of hearing impairment	Assistive listening devices	First language
Barry	23	Male	3rd	Birth	Profound (L & R)	Cochlear implant (R)	English
Merle	21	Female	3rd	Birth	Moderate (L & R)	None	Afrikaans
Paul	24	Male	3rd	Birth	Profound (R)	BTE Hearing aid (R)	Afrikaans
Astrid	24	Female	4th	*L* = 4 yr *R* = 8 yr	Profound (L & R)	Cochlear implant (R)	English
Colin	20	Male	1st	Birth	Moderate (L & R)	BTE Hearing aid (R)	Afrikaans
Stewart	20	Male	1st	Birth	Severe (L & R)	BA Hearing aid (L & R)	Afrikaans
Noelene	19	Female	1st	*L* = 2 yr *R* = 10 yr	Profound (L & R)	Cochlear implant (R)	English

*Source*: Bell 2013

*L*, left; *R*, right; BTE, Behind-the-ear; BA, Bone anchored.

### Data generation methods

In-depth interviews were used to generate the data. I arranged an initial meeting with each student, a ‘get-to-know-each-other’ session, to build a relationship. At this meeting, I explained the nature and aim of the research project, requested their participation and asked them to complete a biographical questionnaire, which provided a large amount of background information, for example, type of hearing loss, age of onset, use of assistive technologies. A follow-up meeting was scheduled with each student at which the individual in-depth interview took place.

The interviews were conducted in a quiet location on campus, to facilitate barrier-free communication. An interview guide (refer to Box 1) was designed to ensure that all relevant topics were covered during each discussion (Patton 2002). This assisted to ensure that certain themes were explored in depth. Participants were provided with a copy of the interview guide so that they could read the questions as well as listen to them being asked. The interviews were digitally audio-recorded with the written consent of the participants with the field notes taken during the interviews assisting the researcher in formulating new questions or returning to others that required more discussion during that session.

BOX 1 Interview guide.

**Table:** 

Grand tour question	‘Could you please tell me about your experiences as a student with a hearing impairment at this University, thinking back right from when you applied to study …’
Question 1	‘Could you please tell me about various learning support services you may have made use of during the course of your studies?’
Question 2	‘As a student with a hearing impairment, please comment on curriculum accessibility as experienced by you in your course of study.’
Question 3	‘Have you experienced any barriers to learning and if so, how have you attempted to overcome these barriers?’
Question 4	‘What, in your opinion, would you regard as critical factors for academic success as a student with a hearing impairment studying at this university?’
Question 5	‘What is your view on including students with disabilities into mainstream education?’

*Source*: Bell (2013)

### Making meaning of the data

Interviews were transcribed and then ATLAS.ti was used to code the data, form categories and themes and build network views (Charmaz 2006; Friese 2012). I followed the grounded theory coding process as explained by Charmaz (2006:46) as it allowed theory to be ‘built’ from the data. This process involved an:

- Initial coding phase: involves naming each word, line or segment of data.
- Focused coding phase: uses the most significant or frequent initial codes to sort, synthesise, integrate and organise large amounts of data.
- Theoretical coding phase: a sophisticated level where the theoretical codes specify possible relationships between categories developed during the focused coding.

Memos (within ATLAS.ti) were used throughout the data transformation process to assist with data interpretation while transforming the data. The measures used to ensure trustworthiness of the data were crystallisation (Richardson 2000:934), member checks (Holloway 1997:160), peer review (Merriam 2009:219–220) and an audit trail (Silverman 2010).

## Ethical considerations

Students with a hearing impairment, or any other type of disability, are regarded as a vulnerable group (Shargorodsky *et al*. 2010). For this reason, utmost care was taken to comply with ethical procedures. In this study, the rights, needs, values and desires of the participants were fully respected. Permission was obtained from the study university’s Ethical Clearance Committee to conduct the research, and the following ethical arrangements were taken into consideration: informed consent, anonymity, and confidentiality and protecting the participants from any harm.

## Research findings and interpretive discussion

This section will present the findings and provide an interpretive discussion around each.

### Findings

Although the research goal of the larger study (Bell 2013) focused on the overall academic experiences of students with hearing impairment using oral communication at university, the focus of this article is on the most significant finding, namely that all student participants were identified as having a hearing impairment rather than a D/deaf identity, which formed part of their cultural paradigm. Linked to this was their unwillingness to disclose their hearing loss, which impedes access to support and possibly affects their eventual academic success. The findings are thus discussed by centring on a Hearing/Deaf identity cultural paradigm and disclosure of hearing impairment.

### Hearing/deaf identity cultural paradigm

Having a hearing or D/deaf identity cultural paradigm refers to how the participants in this study perceived their self-identity. As asserted by Thomas (2002:72), there is a grey zone between a normal and a disabled bodily state, which raises the question of identity. All participants in this study identified strongly with having a hearing identity, taking the hearing world as their ‘reference point for normality and the Deaf world for abnormality, disability and deviance’ (Glickman & Carey 1993:276). Embracing a culturally hearing identity refers to the dominant hearing understanding of deafness as a medical pathology, as per Glickman and Carey’s (1993) DIDS. Culturally hearing persons, such as the participants in this study, value oral means of communication such as speech-reading, lip reading, use of residual hearing as well as fitting comfortably within the larger hearing world.

Participants in this study claimed self-identities that shift the focus away from the disability. This is in line with Johnstone’s (2004) view of disability as an identity being a personal construction or a purposive attempt to making meaning of oneself in the world. If forced to disclose their hearing impairment, they would assume an overcompensating identity in order to cope with the notion of being classified ‘disabled’. This is clearly evident as shared by Astrid and Merle,

‘With me having a disability in the first place, I don’t see myself as disabled, I don’t see myself as being part of … [disabled] society. It has always been like that so … I was never regarded as someone who was deaf … even though I was deaf, I still went to school normally, they treated me normally and it wasn’t that I was isolated from the rest of the world, so I was part of it, the teachers were supportive and the students also.’ (Astrid)

‘I really don’t see myself as being disabled … I have never been treated as someone who is deaf … and with me they won’t see immediately, they will assume that I am a normal person. That is how it has always been.’ (Merle)

I would assert that there are three main reasons for the participants in this study assuming a hearing identity: oral communication tradition, previous experience and invisible nature of hearing equipment. The first reason could be that they come from an oral communication tradition where lip- and speech-reading as well as the use of their residual hearing are valued. Except for one student, all seven participants came from hearing families. The participants also only generally interacted with hearing friends and peers, feeling that their self-identity should depend on personal rather than audiological definitions, and consequently contact with hearing peers was valued (Leigh 1999). In such an environment, the hearing world is taken as the reference point for normality and the participants therefore did not view themselves as abnormal or disabled in any way.

This characteristic of normalisation was a recurring theme throughout the data. The participants did not want to be seen as ‘different’, but rather viewed themselves as normal hearing university students. Noelene expressed her self-identity as follows:

‘… and with me they won’t see immediately, they will assume that I am a normal person. That is how it has always been … I have always been a normal student.’ (Noelene)

The stigma of being labelled as disabled was strongly rejected by the participants. Astrid explained her experiences at school as positive as she was not viewed as being disabled, while Paul shared how he tried to cope on his own without disclosing his hearing impairment as he did not want any special concessions. The comments of this participant illustrate the rejection of labels.

‘I had a lot of teachers that would never treat me as a disabled student in the first place, but they would also forget about it [my hearing impairment] sometimes and when I think back now that was really good – that you are not different from anyone else, so just get on with it [life].’ (Astrid)

Part of their hearing identity was taking on the responsibility to fit in and cope in a hearing world in order to gain employment and eventually be economically independent. One of the participants (Barry) mentioned that he viewed the use of oral communication as extremely important and that the use of Sign Language was severely limiting due to special schools being under-resourced and therefore often viewed as having lower academic standards than mainstream public and private schools. None of the seven participants had ever been exposed to Deaf culture or Deaf communities and felt that the use of Sign Language was not supported in higher education or society at large and was therefore never an option for consideration. It also seemed that in a ‘hearing’ academic setting, which does not support the use of SASL, students with hearing impairment do not have a choice, in any event, but to assume a Deaf identity.

The second possible reason for the participants having a hearing identity is previous experience, namely the fact that they all came through mainstream primary and secondary schools, except for one student who attended a special primary school for learners with hearing impairment. In the mainstream environment, they reported that they were not treated as learners with a disability.

Thirdly, because of the often ‘invisible’ nature of a hearing impairment, especially for girls with long hair covering their hearing aids (speech processors), students with hearing impairment are often seen as ‘normal’ as the sensory impairment is not easily visible. In some cases, participants reported that they purposefully hid their hearing instruments in order to avoid being labelled and stigmatised as ‘deaf’ or ‘disabled’. If is often for this same reason, namely to remain ‘invisible’, that students with hearing impairment refuse to make use of any assistive listening devices which could make them ‘identifiable’ or ‘extra-visible’ (Goode 2007).

The issue of identity is critical as students’ self-perception/self-identity and their perception of how others view them play a pivotal role in their interactions with both institutional processes and structures, and this may have important implications for their academic success. For example, if a student with hearing impairment has a self-perception of being ‘normal’ or non-disabled, then his or her interaction with the institutional processes will be as a hearing student, without disclosing or requesting any learning support, potentially resulting in poorer academic results. The outcomes of this study support the findings by Hindhede (2011), indicating that due to the invisibility of hearing impairment, the hearing disability identity is open to perpetual negotiation and, by avoiding confronting their impairment, the students are not able to enjoy full and equal participation in academic life which has the potential to result in poor educational outcomes.

### Disclosure of hearing impairment

Disclosure of hearing impairment refers to the willingness (Department of Labour n.d.) of students to disclose their type of disability either on their university application form, to their lecturers and/or to their peers. In this study, it was found that the willingness of the student participants to disclose their hearing impairment was either purely for administrative purposes or to solicit additional support when encountering specific barriers to learning such as not being able to lip-read when lecturers walk around in the class, noisy classroom environments, etc. Similar reasons for disclosure were also reported in a study by Getzel and Thoma (2008). It seems that the only reason why student participants disclosed on the university application form was due to it being viewed as a ‘legal’ requirement. Had they been given a choice, they would not have disclosed their hearing impairment freely (ignored the section) as clearly explained by Astrid:

‘I had to because it was on the piece of paper obviously, what kind of disability do you have? Are you deaf? So I ticked the ‘deaf’ one.’ (Astrid)

The participants also referred to their hearing impairment as being ‘not so visible’ and they reported that friends often did not realise that they have a hearing impairment due to its ‘invisibility’. As Merle said:

‘Very few of them [friends], because it is not so visible, many people don’t know, some of my friends don’t even know.’ (Merle)

Some disclosed their hearing impairment to their lecturers, but generally only if compelled to do so through circumstances, and one of the participants deliberately used his ‘disabled’ status to secure a place in the university residence. Disclosure at university seems to be a reactive action in most cases, as expressed by Noelene:

‘I never went to tell them of my disability … I feel it is not necessary to tell them unless I have a problem.’ (Noelene)

This phenomenon also made it difficult in the early phases of the research study to identify the student participants for this investigation.

The most logical explanation for participants’ non-disclosure could be linked to their ‘hearing identity’ and their rejection of being labelled or stigmatised as ‘disabled’ (Watson 2002). In South Africa, there are also no positive incentives to encourage disclosure such as the Disabled Student’s Allowance, which is offered to students with disabilities in the United Kingdom. A further reason for participants’ non-disclosure could be linked to the fact that at school it was not necessary to disclose their hearing impairment in order to solicit any particular support as none was available or the additional support was not required due to smaller class sizes or a lack of self-advocacy skills.

It was clear that the participants in this study did not want to define themselves or their relationships with others based on their hearing impairment. They tried to assimilate as much as possible into the hearing world, identified as culturally hearing, and thus chose not to disclose their hearing loss.

## Conclusion and recommendations

Despite increased access for students with hearing impairment into higher education, such students generally remain under-represented, unaccounted for and under-supported; experiencing many barriers and having to develop personalised coping strategies. One reason for this could be linked to their self-identity as the issue of assumed identity plays a crucial role in their personal choice to disclose (or not disclose) their disability which in turn affects their access to teaching and learning support.

All the participants in this study came from hearing families and they made use of spoken language. Factors such as choice of communication, family environment, attending mainstream schools and a focus on the person as opposed to the impairment led the students to assuming a hearing cultural identity rather than a D/deaf cultural identity and electing not to disclose their hearing loss for fear of stigmatisation. In this way, they believed that other people would see them as normal and not as disabled. In addition to assuming a hearing identity, the participants did not self-advocate in order to negotiate for their communication needs (full accessibility in the teaching and learning environment). This is directly related to their hearing self-identity and the level of importance they attached to being invisible as opposed to extra-visible and drawing attention to themselves. The need to blend in and to be seen as normal seemed to be high. Their personal choice of non-disclosure may be detrimental to their academic success as it limits the extent to which the university/disability unit is able to provide appropriate support. If the communication barriers that they experience in the teaching and learning environment become insurmountable or if their personalised coping strategies are insufficient or ineffective, this may lead to poor academic outcomes, affecting their future economic well-being.

These findings offer practical value for the individual, the disability unit staff and the university as a whole:

Suggestions for the individual with a hearing impairment:

- Develop and make use of skills to self-advocate by informing significant role players in their university education of their hearing impairment and be able to negotiate for the necessary accommodations.
- Build effective relationships with lecturers, peers and staff from the disability unit to be able to interact appropriately and confidently and negotiate communication access.

Suggestions for the disability unit staff and the university:

- Communicate to students with hearing impairment the benefits of and reasons for disclosure.
- Ensure that students are fully informed regarding all aspects of supporting the communication and accessibility needs of students with hearing impairment, including up-to-date knowledge of the latest available assistive technologies, and remain sensitive to their needs.
- Ensure that students with hearing impairment have as much knowledge as possible (concerning their rights as students with disabilities and the availability of support) to assist them to make good decisions about their communication and other support needs.
- Have in place clear institutional guidelines concerning disclosure and confidentiality.
- Make available financial and human resources to support the access needs of students with hearing impairment.

The education of students with hearing impairment, using the oral approach for communication at mainstream schools, colleges and universities in South Africa is an under-researched area. The following recommendations for future research are suggested:

- to identify the skills, strategies and awareness necessary for increased disclosure and self-advocacy and
- to identify the specific types of support that students with hearing impairment are receiving at universities throughout South Africa, as well as their experiences, both negative and positive, in relation to these.

It is of vital importance that students with a hearing impairment who have a hearing cultural identity be taught the skill of self-advocacy and self-representation from an early age so that they become comfortable and confident in disclosing their disability and not feel ashamed or stigmatised. In this way, they also learn to self-advocate for their communication accessibility needs, not having to rely on their personal coping strategies but on the support (both technological and human) provided by the institution, the disability unit, their lecturers, their tutors and their peers, which could lead to improved educational outcomes.
